# Natural *β*-Carotene Production by *Blakeslea trispora* Cultivated in Spanish-Style Green Olive Processing Wastewaters

**DOI:** 10.3390/foods10020327

**Published:** 2021-02-04

**Authors:** Eugenia Papadaki, Fani Th Mantzouridou

**Affiliations:** Laboratory of Food Chemistry and Technology, Department of Chemistry, Aristotle University of Thessaloniki, 54124 Thessaloniki, Greece; epapadaki@chem.auth.gr

**Keywords:** natural *β*-carotene, *Blakeslea trispora*, table olive wastewaters, hydroxytyrosol, tyrosol

## Abstract

In the current research, the potential of Spanish-style green olive processing wastewaters (lye and washing waters) exploitation toward natural *β*-carotene production by *Blakeslea trispora* was tested for the first time. Mating culture generated by the joint cultivation of the heterothallic fungal strains ATCC 14271 and 14272 in the non-sterile lye and washing waters was able to grow, achieving the phytotoxic hydroxytyrosol degradation by 57.3% and 66.8%, respectively. However, the low sugar and nitrogen content of the streams did not favor carotenogenesis. Alternatively, in the nutrient-enriched effluents, a notable quantity of *β*-carotene was produced, accounted for 61.2 mg/L (lye) and 64.1 mg/L (washing waters) (82–88% of total carotenoid content). Above all, enriched streams had a noteworthy stimulating effect on the *β*-carotene synthesis, because both the maximum *β*-carotene yield per volume of enriched effluents and specific *β*-carotene production rate were higher when compared with the respective values obtained from trials with synthetic reference medium without added effluents. Hydroxytyrosol and tyrosol showed high stability during the non-sterile process for *β*-carotene production by *B. trispora* grown in the enriched effluents. This finding strengthens the potential toward the generation of multiple high-value products, which could lower the natural *β*-carotene production costs.

## 1. Introduction

*β*-Carotene is a natural functional pigment, mostly found in biological systems as an all-*trans* isomer [1]. This carotenoid is widely used in the production of foods, beverages, animal feed, cosmetics, and pharmaceuticals. The industrial importance results from its provitamin A, antioxidant, and immunomodulatory activity as well as its protective effect against cancer and cardiovascular diseases. Additionally, *β*-carotene serves as a coloring agent, providing various shades of yellow, orange, and red [2]. As the world is currently facing the negative impacts of the coronavirus disease 2019 (COVID-19) pandemic on global health and the economy, the adoption of balanced nutritional patterns has become a necessity. In this direction, the uptake of *β*-carotene and its derivatives as micronutrients during quarantine may boost the immune response and resistance of the human body to viral infections [3].

The commercial market value of *β*-carotene was estimated to be $314.14 billion in 2020 and is expected to reach $380.37 billion in 2025 at an annual growth rate of 3.9% from 2020 to 2025 [4]. Nowadays, *β*-carotene is mainly produced via chemical synthesis to meet the demand in various sectors. However, increasing consumer preference for natural and clean label products has triggered the development of the natural carotenoids sector [5]. Plant sources of *β*-carotene, with the most common being carrots, are not expected to address the current demand for technological purposes due to low yields, seasonal and geographical variations as well as high raw material cost [6]. In this context, microorganisms have been proved to be a valuable source of *β*-carotene despite challenges and limitations on scale-up strategies, manufacturing costs, and regulatory approval processes [7]. Among them, the fungus *B. trispora* is of primary industrial interest as a source of *β*-carotene for commercial exploitation [7]. Its status is related to the fact that *β*-carotene from *B. trispora* is the first authorized microbial food colorant in the European Union (E160a(ii)) [8]. From a technological perspective, *B. trispora* can achieve an efficient and high-yielding synthesis of all-*trans β*-carotene at the expense of other structurally related carotenoids when mycelia of the two mating types, namely plus (+) and minus (−) mating types, are co-cultivated [9,10,11,12]. Each mating type develops zygophores that grow toward each other and produce progametangia. The latter separate into terminal gametangia that fuse to form the zygospores, leading to the accumulation of *β*-carotene [13]. Driven by the quest for sustainability, the mating strains *B. trispora* ATCC 14271 (+) and ATCC 14272 (−) have gained the interest of investigators because of their ability to upgrade a variety of inexpensive substrate constituents or even industrial waste products (e.g., beet molasses, cheese whey, crude vegetable oils, crude glycerol, cabbage waste, watermelon husk, peach peels, waste cooking oils) into the valuable end product *β*-carotene, moderating at the same time environmental pollution and reducing the manufacturing cost [14,15,16,17,18,19].

To develop innovative sustainability processes for *β*-carotene production by *B. trispora*, our research activity was focused on the exploitation of Spanish-style green olive processing wastewaters as a fermentation feedstock. Particularly, polluting lye and washing water effluents attracted our interest considering that these streams are generated in a short space of time (~1 month) at high volumes (~5 million m^3^) on a global scale [20,21], with the main contributors being Spain, Egypt, Turkey, Algeria, and Greece [22]. The absence of specific legislative measures for the effective treatment of these streams constitutes a significant environmental issue that the table olive industry is facing. Although effluents storage in evaporation ponds is the main applied treatment so far, this approach involves several risks related to the contamination of the terrestrial ecosystem and the release of fetid gasses [20]. To address the above problem, several studies have been carried out toward the effective detoxification of the streams through the eco-friendly and inexpensive biological treatment with activated sludge, fungi (*Aspergillus niger*, *Geotrichum candidum*, *Trichoderma harzianum*, white-rot fungi) or microalgae (*Nannochloropsis gaditana*) [20,23,24]. In recent investigations from our group [21,25], these effluents have been valorized for the microbial production of citric acid by *A. niger* with the integrated co-production of hydrolytic enzymes with higher commercial value. The findings from the techno-economic analysis highlighted that the high-value co-products improved the process economics. The latter was the driving force to carry out experiments for the valorization of these effluents through *β*-carotene production by *B. trispora*. To our knowledge, no attempts to exploit the effluents for carotenoid production via microbial fermentation appear in the literature so far because of the toxicity and nutrient unbalance of the streams [20,26].

The current study aimed to investigate the dynamics of natural *β*-carotene production by *B. trispora* growing in lye and washing waters from Spanish-style green olive processing. Biochemical and kinetic interpretations concerning fungal growth and carotenoid production in non-sterile effluents without and after supplementation with nutrients were considered and discussed. The evolution of the high-value compounds hydroxytyrosol and tyrosol during the biological treatment was also determined to support further the valorization strategy.

## 2. Materials and Methods

### 2.1. Reagents

All-*trans β*-carotene (for biochemistry, purity 97%) was purchased from Merck (Darmstadt, Germany). Hydroxytyrosol, tyrosol, caffeic acid, luteolin-7-*O*-glucoside, *p*-coumaric acid, and oleuropein were obtained by Extrasynthese (Genay, France). Phosphoric acid and butylated hydroxytoluene (BHT) were from Sigma-Aldrich (Steinheim, Germany). Folin–Ciocalteu reagent and HCl as well as HPLC grade acetonitrile, acetone, *n*-hexane, ethyl acetate, and methanol were purchased from Chem-Lab NV (Zedelgem, Belgium). D-Glucose monohydrate, casein acid hydrolysate, yeast extract, L-asparagine, KH_2_PO_4_, MgSO_4_·7H_2_O, thiamine hydrochloride as well as emulsifiers Span 20 and Tween 80 were from Scharlau Chemie S. A. (Barcelona, Spain). D-Fructose was obtained by Panreac (Barcelona, Spain), while LCK 338 cuvette test was from Hach Lange (Düsseldorf, Germany). Potato dextrose agar (PDA) was supplied by Lab M Limited (Heywood, UK). All the other reagents and solvents of appropriate grade were purchased from various producers.

### 2.2. Feedstocks

Fresh lye and washing water effluents from Spanish-style processing of green olives (cv. Chalkidiki) (2016-2017 processing season) were supplied by an industrial plant located in Chalkidiki (Northern Greece). Sampling was from three different tanks processed in parallel. Lye (20 L) was collected just after olive treatment with 2% (*w/v*) NaOH aqueous solution for 11 h, while washing waters (20 L) were obtained after two water changes at 8 h and 16 h. Then, the streams were stored immediately at −20 °C until use.

### 2.3. Microorganisms

The microorganisms used in this study were *B. trispora* ATCC 14271, mating type (+), and *B. trispora* ATCC 14272, mating type (−). Both strains were donated by the German chemical industry BASF Aktiengesellschaft (Ludwigshafen, Germany). The strains were grown separately on PDA petri dishes at 26 °C for 5 d to prepare the spore inoculum. Stock cultures were stored as spore suspensions at −80 °C in cryotubes containing 20% glycerol.

### 2.4. Submerged Fermentation

Experiments were carried out in 250 mL Erlenmeyer flasks containing 50 mL of non-sterile lye or washing waters without and after supplementation with the following ingredients (g/L): glucose (50), casein acid hydrolysate (2), yeast extract (1), L-asparagine (2), KH_2_PO_4_(1.5), and thiamine hydrochloride (0.005). Dispersed growth of *B. trispora* was obtained by the addition of 1% (*w/v*) Span 20 and 0.1% (*w/v*) Tween 80 [9]. The initial pH value of the streams was adjusted to 7 with HCl (37%, *w/w*). Each flask containing the effluents was inoculated with both (+) and (−) mating types of *B. trispora*. Appropriate volumes of spore suspensions were prepared to obtain about 2 × 10^5^ spores/mL of each strain in the culture medium. The spore concentration in the suspension was determined using a Neubauer hemocytometer (BlauBrand, Wertheim, Germany). Liquid cultures were incubated at 25 °C for 6 d on a rotary shaker (KS 4000i control, IKA, Wilmington, NC) operating at 160 rpm. Non-inoculated effluents, as well as a synthetic medium containing the above ingredients plus MgSO_4_·7H_2_O (0.5 g/L) in sterile distilled water [9], were incubated under the same conditions and used as reference substrates. At specific time intervals, three flasks were removed from the incubator and each fermentation broth (liquid and biomass) was analyzed.

### 2.5. Determination of Total Dry Biomass Content

To determine total dry biomass content (g/L), aliquots in triplicate of each homogeneous fermentation broth were filtered under reduced pressure (Pump V-700, Büchi, Flawil, Switzerland) through a Whatman 1 filter paper, rinsed twice with distilled water, and dried at 105 °C until constant weight.

### 2.6. Determination of Total Suspended Solids

Total suspended solids (g/L) in the wastewater samples were determined in triplicateaccording to the procedure described in Standard Methods [27].

### 2.7. Determination of Soluble Sugar Content

The wastewater samples were suitably diluted with distilled water and filtered through a 0.45 μm PTFE filter (Waters, Milford, MA). Then, the effluent filtrates were subjected to high-performance liquid chromatography (HPLC) analysis for the determination of glucose and fructose. The HPLC system was equipped with a LC-10Advp pump (Shimadzu, Kyoto, Japan) and a RID-6A refractive index detector (Shimadzu). The separation was achieved on an Agilent HI-plex H (Agilent Technologies, Santa Clara, CA, USA) column by isocratic elution with ultrapure water at 65 °C. The flow rate was 0.5 mL/min and the injection volume 10 μL. Each sample was analyzed in triplicate. Quantification was performed by linear regression analysis using external calibration curves for glucose (g/L) and fructose (g/L). Total soluble sugar content (g/L) in the effluents was determined spectrophotometrically by the phenol-sulfuric acid method and expressed as glucose equivalents (g/L) [21].

### 2.8. Determination of Total Nitrogen Content

Total nitrogen content (mg/L) in the wastewater samples was assessed in triplicate by the persulfate digestion method using a total nitrogen kit LCK 338 and a DR 3900 spectrophotometer (Hach Lange) [26].

### 2.9. Determination of Metals

The preparation of the wastewater samples for metal analysis was accomplished via drying at 105 °C followed by acidification with HCl (6 N), burning over Bunsen burner, and incineration at 550 °C. The ash was suitably diluted with HCl (6 N) and the solution was analyzed on a Perkin-Elmer Optima (model 3100 XL) axial viewing atomic emission spectrometer with inductively coupled plasma. The system was equipped with a free-running radio frequency generator (40 MHz, 1500 W incident power) and a segmented-array charge-coupled detector. A fassel-type torch was employed with a nebulizer argon flow rate of 0.80 L/min. A cyclonic-type spray chamber and a Babington-type nebulizer were used for the nebulization. The sample uptake flow rate was 2 mL/min. The spectral wavelengths were set at 213.857 nm (Zn), 239.562 nm (Fe), 257.610 nm (Mn), 280.271 nm (Mg), and 324.752 nm (Cu). The analysis of each sample was carried out in triplicate. Quantification of each metal was performed using standard curves and calculated by linear regression analysis (mg/L or % on a dry mass basis) [21].

### 2.10. Determination of Total Carotenoid and β-Carotene Content

#### 2.10.1. Extraction of Carotenoids and Determination of Total Carotenoid Content

The carotenoids were extracted from fungal cells through freezing and thawing with the aid of liquid nitrogen, followed by manual grinding in the presence of quartz sand until complete cell lysis occurred. Complete cell destruction was monitored by a phase-contrast microscope (Nikon E 200, Melville, NY, USA) using 100× magnification for image analysis (Matrox Inspector version 3.0, Matrox Electronic Systems Ltd., Dorval, QC, Canada). For carotenoid extraction, the cell suspension was mixed with an equal volume of acetone containing BHT (0.1%, *w*/*v*) to prevent carotenoid oxidation. Extraction was carried out in vessels covered with aluminum foil and sealed with a vented cap, away from light exposure, to protect carotenoids from photodegradation and isomerization during extraction. The extraction was performed in triplicate on a rotary shaker (160 rpm, 30 °C, 30 min) until the suspension became colorless. The colored organic layer was then centrifuged (10,000× *g*, 10 min) to remove mycelia cells, passed through a water-free Na_2_SO_4_ layer, and evaporated to dryness under vacuum at ~35 °C (Büchi). The dry carotenoids extract was dissolved in *n*-hexane and used for the spectroscopic determination of total carotenoid content at 450 nm. Results were expressed as *β*-carotene equivalents (mg/L broth or mg/g dry biomass).

#### 2.10.2. RP-HPLC Analysis of Cellular *β*-Carotene Content

Carotenoid dry extracts (Section 2.10.1) were suitably diluted in acetone and filtered through a 0.45 μm PTFE filter (Waters) before chromatographic analysis. Then, the extracts were analyzed on a reversed-phase HPLC (RP-HPLC) system equipped with a LC-20AD pump (Shimadzu, Kyoto, Japan) and a SPD-10AV UV-VIS detector (Shimadzu). The column used was C_18_ (250 mm × 4.6 mm i.d., 10 μm) (Macherey-Nagel, Düren, Germany) and the injection volume was 10 μL. The samples were eluted with acetone/acetonitrile (60/40, *v*/*v*) at an isocratic flow rate of 1.2 mL/min. Each extract was analyzed in triplicate. The identification and the purity of all-*trans β*-carotene peak were confirmed by comparison of its retention time and spectral characteristics with those of the authentic standard and published information as well as by peak spiking with the standard. Quantification of all-*trans β*-carotene content (mg/L broth or mg/g dry biomass) was accomplished with the aid of an external calibration curve in the range of 2.0–30 ng/µL. The response was detected at 453 nm [11]. The percent of *β*-carotene, *γ*-carotene, and lycopene content was calculated based on the total area of all peaks detected at 453 nm. The spectra of carotenoid peaks were recorded in the region 380–700 nm using a RP-HPLC system equipped with a SCM1000 vacuum membrane degasser (Thermo Separation Products Inc., San Jose, CA, USA), a P4000 pump (Thermo Separation Products Inc.), a Midas autosampler (Spark, Emmen, The Netherlands), and a multiple-wavelength UV 6000 LP diode array detector (DAD; Thermo Separation Products Inc.). The separation was performed using the same column and elution conditions described above.

### 2.11. Determination of Polar Phenolic Compound Content

#### 2.11.1. Extraction of Phenolic Compounds and Determination of Total Polar Phenol Content

The polar extracts of the wastewater samples were obtained by liquid-liquid extraction. Before the extraction, the pH value of the effluents was adjusted to 2 with HCl (6 N). Then, an equal volume of petroleum ether was added to the streams. The mixture was vortexed (1 min) and centrifuged (3500× *g*, 10 min) for the removal of lipid traces. Next, the aqueous phase containing the polar phenols was collected and extracted three times with an equal volume of ethyl acetate by using vortex (1 min) and centrifugation (3500× *g*, 10 min). The organic extract was evaporated to dryness under vacuum at ~35 °C (Rotavapor, Büchi). The dry extract was dissolved in methanol and filtered through a 0.45 μm PTFE filter (Waters). Total polar phenol (TPP) content in the extracts was estimated spectrophotometrically by the Folin–Ciocalteu assay and expressed as caffeic acid equivalents (mg/L). The determination was carried out in triplicate [26].

#### 2.11.2. RP-HPLC Analysis of Phenolic Compounds

The methanolic extracts (Section 2.11.1) were analyzed by RP-HPLC. The system consisted of a SCM1000 vacuum membrane degasser (Thermo Separation Products Inc.), a P4000 pump (Thermo Separation Products Inc.), a Midas autosampler (Spark), and a UV 6000 LP DAD (Thermo Separation Products Inc.) connected in series with a SSI 502 fluorescence detector (FLD; Scientific Systems Inc., State College, PA, USA). The column used was C_18_ (250 mm × 4.6 mm i.d., 5 μm) (Supelco, Sigma-Aldrich). The elution system consisted of 0.2% aqueous phosphoric acid (solvent A) and acetonitrile (solvent B). The gradient elution for solvent B was 10% (0–1 min), 10–20% (1–10 min), 20–50% (10–43 min), 50–95% (43–48 min), 95% (48–52 min), 95–10% (52–60 min). The flow rate was 0.5 mL/min and the injection volume 10 μL. Each extract was analyzed in triplicate. The identification and the purity of each peak were based on peak purity function of the DAD, spectral characteristics, retention time, peak spiking with authentic standards, fluorescence (FL) detection, and literature. Ultraviolet (UV) spectra of the peaks were recorded in the region 220–500 nm online. Quantification of individual phenolic compounds (mg/L) was performed using external calibration curves for hydroxytyrosol (FL exc 280 nm/em 320 nm and UV 280 nm), tyrosol (FL exc 280 nm/em 320 nm), oleuropein (UV 240 nm), caffeic acid (UV 280 nm), luteolin-7-*O*-glucoside (UV 280 nm), and *p*-coumaric acid (UV 280 nm) in the range of 5–120 ng/µL, 2–80 ng/µL, 5–100 ng/µL, 0.2–10 ng/µL, 0.5–20 ng/µL, and 0.5–20 ng/µL, respectively. In addition, hydroxytyrosol and oleuropein were used for methoxy derivative of hydroxytyrosol and oleoside-11-methyl ester quantification at UV 280 nm and 240 nm, respectively, multiplied by their molecular weight ratio (168/154 and 404/540, respectively) [26].

### 2.12. Determination of Growth and β-Carotene Production Kinetics

Among the different models (Logistic, Gompertz, and Richards) tested, the modified Logistic model was the one having the highest coefficient of determination and, thus, it was used to describe the growth of *B. trispora* (Equation (1)) and *β*-carotene production (Equation (2)) in the culture medium [28,29]:

(1) $$X=\;\frac{X_{m}}{1\;+\exp\left[\frac{4\mu_{m}}{X_{m}}\left(\lambda_{B}-t\right)+2\right]}$$

where X is the dry biomass content (g/L broth) at time t (h); *X*_m_ is the maximum value of dry biomass content (g/L broth), *μ*_m_ is the maximum specific growth rate (1/h); *λ*_B_ is the growth lag time (h), and t is the time (h).

(2) $$P=\;\frac{P_{m}}{1\;+\exp\left[\frac{4R_{m}}{P_{m}}\left(\lambda_{CR}-t\right)+2\right]}$$

where P is the *β*-carotene content (mg/L broth) at time t (h); *P*_m_ is the maximum value of *β*-carotene content (mg/L broth); *R*_m_ is the maximum specific *β*-carotene production rate (1/h); *λ*_CR_ is the *β*-carotene production lag time (h), and t is the time (h).

### 2.13. Statistical Analysis

Each dataset was submitted to the general linear model procedure followed by descriptive statistics to test the normal distribution of the residuals, using SPSS v. 20.0 software (SPSS Inc., Chicago, IL, USA). The results obtained by the Shapiro–Wilk test revealed a significance level of greater than 0.05 for each sample data, verifying normality (i.e., the null hypothesis of non-normal distribution was rejected). Then, statistical comparisons of the mean values were carried out either by one-way ANOVA, followed by Duncan’s test, or by Student’s *t*-test (*p* < 0.05 significance level) (SPSS). The kinetic models were fitted to the experimental data with Microsoft Excel spreadsheet using Solver function (Microsoft Corp., Redmond, WA, USA) in order to find the values of the kinetic parameters that result in the minimum level for sum chi-squared.

## 3. Results and Discussion

HPLC analysis of individual sugars in lye and washing waters revealed the presence of glucose (3.0 g/L ± 0.0 g/L and 2.5 g/L ± 0.1 g/L, respectively) and fructose (3.1 g/L ± 0.0 g/L and 2.6 g/L ± 0.0 g/L, respectively). In addition, nitrogen content was 93.8 mg/L ± 2.8 mg/L and 126.0 mg/L ± 3.5 mg/L in the respective streams. Because the amount of carbon and nitrogen sources in the effluents may not be satisfactory to support fungal growth and carotenogenesis [9], nutrient-enriched lye and washing waters were included in the present study. Selection of the type and the level of ingredients to prepare the enriched streams (see Section 2.4) was based on the relevant literature data for nutrients required in synthetic medium to support B. trispora growth and carotenoid production [9]. The evaluation of fungal growth and carotenogenesis in the fermentation media containing table olive processing wastewaters as process water for β-carotene production by B. trispora will provide a strong basis for the design of future integrated biorefinery approaches that aim to reduce freshwater requirements and external nutrients by mixing the wastewaters with multiple feedstocks [21]. Experiments were also conducted using the synthetic medium as a reference one. Considering that the level of Mg in lye (51.9 mg/L ± 2.0 mg/L) and washing waters (46.4 mg/L ± 1.7 mg/L) was sufficient for the performance of B. trispora [9], this metal was only added to the synthetic medium. However, the proposed approach cannot preclude any potential negative impact of the effluents on the fungal growth and carotenogenesis due to their extreme physicochemical characteristics (high levels of NaOH and suspended solids), as well as the presence of phenolic compounds with antimicrobial properties [26]. To the best of our knowledge, no related data exists in the literature. In the current research, data concerning fungal growth and carotenoid production in non-sterile effluents and the enriched ones are presented in Section 3.1 and Section 3.2, while the evolution of polar phenolic compounds in the effluents throughout fermentation is discussed in Section 3.3.

### 3.1. Kinetics of Microbial Growth

**Figure A1:**
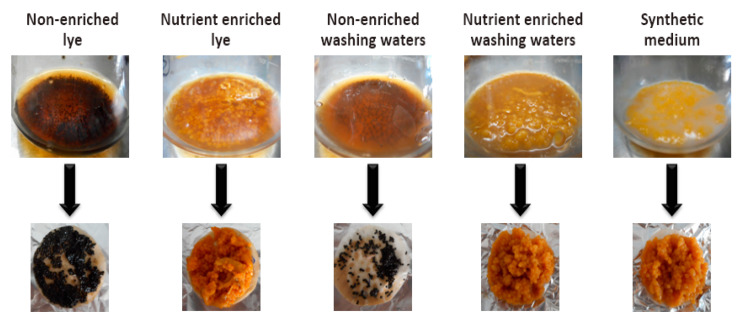
Macroscopic appearance of *Blakeslea trispora* in non-sterile lye and washing waters from Spanish-style green olive processing, without or after enrichment with nutrients, as well as in synthetic medium (fermentation time: 6 days).

To investigate the effect of lye and washing waters upon fungal growth, kinetic studies were carried out in the effluents without and after supplementation with nutrients. The modified Logistic model was fitted to the experimental data of dry biomass content for *B. trispora* in order to describe the growth kinetics in the different fermentation substrates (Table 1). The goodness of fit was high (*R*^2^ = 0.982–0.994) in all substrates, indicating that the three-parametric non-linear regression model can satisfactorily describe the mycelial growth. Unsurprisingly, the values of the growth kinetic parameters in the streams without supplementation with nutrients were significantly lower (*X*_m_ < 4 g/L broth and *μ*_m_ < 0.03 1/h) than those in the nutrient-enriched ones (*X*_m_ > 13 g/L broth and *μ*_m_ > 0.22 1/h). This is associated with the nutrient-limiting conditions in the former case, in the response of which spherical black-colored pellets and spores were developed. On the other hand, the growth of *B. trispora* in the enriched effluents promoted the formation of orange-colored large pellets and dispersed mycelia (Figure A1 in Appendix A). Interestingly, the fungal growth parameters showed improvement in the enriched effluents compared with those in the synthetic medium as indicated by the 1.3- to 1.9-fold higher values of *X*_m_ (19 g/L broth and 14 g/L broth in enriched lye and washing waters, respectively) and *μ*_m_ (0.234 1/h and 0.223 1/h in enriched lye and washing waters, respectively) in the former case. This must have been related to the fact that lye and washing waters contain various metals, such as Fe (11.8 mg/L ± 1.7 mg/L and 9.9 mg/L ± 1.6 mg/L, respectively), Cu (2.2 mg/L ± 0.1 mg/L and 1.8 mg/L ± 0.1 mg/L, respectively), Zn (1.0 mg/L ± 0.1 mg/L and 0.8 mg/L ± 0.1 mg/L, respectively), and Mn (0.6 mg/L ± 0.1 mg/L and 0.5 mg/L ± 0.1 mg/L, respectively) which can act as fungal growth factors. Particularly, metals positive effect on biomass formation is associated with their ability to catalyze redox reactions, activate enzyme-systems and/or constitute a part of the enzyme molecules in the fungal cells [30]. For example, Mn is indispensable for the function of metalloproteins (e.g., DNA, RNA, oxidoreductases), while Fe, Cu, and Zn affect the protein structure and act as enzyme-catalysts (e.g., superoxide dismutase, metallothionein) [31]. Considering also that *B. trispora* ENA genes can generate Na^+^-efflux ATPases toward the induction of Na^+^ uptake for cell function [32], the considerably higher level (1.4-fold) of *X*_m_ in nutrient-enriched lye than in nutrient-enriched washing waters could be attributed to the increased Na content in the former [20]. In the only available literature data for the growth kinetics of *B. trispora*, the level of *μ*_m_ calculated by the Baranyi model (0.26 1/h) in glucose-based synthetic medium containing corn steep liquor [12], a by-product of corn wet-milling rich in amino acids, vitamins, and minerals, was comparable to those obtained in the nutrient-enriched table olive processing wastewaters of the current study using the modified Logistic model. This finding strengthens the exploitation of the streams as effective substrates for culturing *B. trispora*.

The lag time (*λ*_B_) of the fungal biomass formation considerably varied between the different fermentation substrates. The fastest growth was recorded in the nutrient-enriched washing waters (10 h) followed by the synthetic medium (14 h). Notably, biomass formation showed a delay in the lye supplemented with nutrients (37 h). A contributor to this phenomenon could be the higher level of suspended solids in the lye than in the washing waters (5.0 g/L ± 0.2 g/L vs. 3.4 g/L ± 0.1 g/L), which can hamper the efficient assimilation of nutrients through turbidity increase and dissolved oxygen concentration reduction [26]. On the other hand, the absence of growth factors from the non-enriched washing waters resulted in the slowest biomass production (46 h). The nutrient deficiency in the stream might also intensify the inhibitory effect of the polar phenolic compounds (TPP content of 715.3 mg/L ± 13.6 mg/L) on the fungal growth. Previous data have highlighted the antifungal activity of olive mill wastewater polar extract rich in phenolic compounds against *A. niger* [33], which is possibly associated with plasma membrane destruction [34]. The 2-fold lower value of TPP content in the non-enriched lye (414.7 mg/L ± 9.1 mg/L) than in the washing waters must have favored the quicker adaptation of *B. trispora* in the former (26 h). Interestingly, the *λ*_B_ value found for the non-enriched lye is similar to that reported in the only available kinetic model study for the cultivation of *Β. trispora* in a glucose-based synthetic medium enriched with refined olive oil (27 h) [12] that is practically free of olive phenolics. Thus, the above findings would be an indication of the *B. trispora* tolerance to the different levels of phenolic compounds in the target waste streams. Nevertheless, synergistic effects may also exist between the above factors.

### 3.2. Kinetics of β-Carotene Production

As shown in Table 2, carotenoid synthesis was not favored in the non-enriched lye and washing waters. On the other hand, the growth of *B. trispora* in the nutrient-enriched effluents resulted in the maximum yield of total carotenoids per volume of substrate, which was 84 mg/L (Table 2), providing the orange color to the fungal biomass (Figure A1 in Appendix A). Carotenoids accumulated at 4.84 mg/g and 5.95 mg/g of dry biomass in nutrient-enriched lye and washing waters, respectively. Among them, *β*-carotene accounted for 82–88% of total carotenoid content (Table 3), specifically 61.2 mg/L (or 3.53 mg/g of dry biomass) and 64.1 mg/L (or 4.57 mg/g of dry biomass) in nutrient-enriched lye and washing waters, respectively (Table 2). In the course of RP-HPLC separation of intracellular carotenoids, a satisfactory separation of three peaks was evidenced at 453 nm. All peaks corresponded to carotenoids based on spectral characteristics in the visible region with regards to published information [11]. All-*trans β*-carotene identification in the effluent extracts was confirmed by comparison of its retention time and spectral characteristics (*λ*_max_ at 429 nm, 452 nm, and 478 nm) with those of the authentic standard analyzed under the same conditions as well as by peak spiking with the standard. The remaining components of total carotenoids were *γ*-carotene (5–9%) and lycopene (8–9%) (Table 3). Noticeably, the above values for total carotenoid and *β*-carotene contents were up to 1.5-fold higher than those recorded in the synthetic medium. This finding must have been associated partially with the presence of metals (such as Fe and Cu) in the effluents that are known to enhance *β*-carotene production through the stimulation of glucose catabolism, trisporic acid formation, and mevalonate kinase activity, providing sufficient pools of carotenoid precursors [35]. To our knowledge, published data concerning the integration of waste streams generated from the manufacturing process of table olives into the production of microbial carotenoids do not exist. The 17-fold higher total yield of carotenoids per volume of the substrate in the current study compared to that in the two-phase olive mill waste-based media by the yeast *Rhodotorula mucilaginosa* (5–7 mg/L) verify the pros of the proposed fermentation process [36]. Moreover, *β*-carotene production by *B. trispora* using other food industry liquid wastes such as beet molasses and deproteinized whey is reported to be of similar size but only after appropriate treatment (enzymatic hydrolysis of disaccharides and sterilization), highlighting further the advantage of the proposed process [14].

Predictive modeling of *β*-carotene synthesis by *B. trispora* can be used to predict the accumulation behavior of the compound in the proposed system and provide insight into the microbial productivity in Spanish-style green olive processing wastewaters. Although this approach has been used effectively for the carotenogenic alga *D. salina* [29], relevant studies for *B. trispora* do not exist yet. Thus, the kinetic parameters of *β*-carotene production (*P*_m_, *R*_m_, *λ*_CR_) were calculated in the synthetic medium as well as in the nutrient-enriched streams by the modified Logistic model (Table 4). The three-parametric non-linear regression model was successfully fitted to the experimental data (*R*^2^ = 0.978–0.999) and can be adequately used as a tool to monitor carotenogenesis in fungal cells. Specifically, the value of *P*_m_ in both streams was 1.6-fold higher than the one detected in the synthetic medium, verifying the positive effect of table olive processing wastewater constituents (e.g., metals) on *β*-carotene production, in line with our suggestion above. Therefore, *R*_m_ level in the synthetic medium (0.8 1/h) was 2.4- and 1.7-fold lower than in the lye (2.0 1/h) and washing waters (1.4 1/h), respectively. Notably, carotenogenesis was delayed in the nutrient-enriched lye (*λ*_CR_ = 47 h) compared to the nutrient-enriched washing waters (*λ*_CR_ = 31 h). This must have been related to the 1.5-fold lower level of suspended solids in the washing waters than in the lye, which can hinder the microbial growth as previously discussed (see Section 3.1). On the other hand, the absence of suspended solids, as well as phenolic compounds with antifungal activity from the synthetic medium, resulted in the accelerated *β*-carotene production in the latter (*λ*_CR_ = 26 h).

### 3.3. Changes in Individual Phenolic Compounds

Among the organic molecules existing in lye and washing waters, particular attention should be given to the phenolic compounds because the latter are related to the streams phytotoxic properties and are not easily biodegradable [26]. On the other hand, the prospect of recovering hydroxytyrosol and tyrosol, two high-value compounds with a wide spectrum of biological activities toward human health protection, seems an interesting alternative [37,38,39]. Thus, it was considered useful to evaluate the ability of *B. trispora* to metabolize the respective molecules. To the best of our knowledge, no related data exists in the literature.

As indicated in Section 3.1, before the biological treatment, the TPP content in the polar extract of lye and washing waters was 414.7 mg/L and 715.3 mg/L, accordingly. The RP-HPLC profile at UV 280 nm and using fluorescence detection system (exc 280 nm/em 320 nm) indicated the presence of the phenolic/secoiridoid compounds presented in Table 5 and Table 6. Hydroxytyrosol was the main phenolic compound, corresponding to 135.9 mg/L in lye and 395.7 mg/L in washing waters. In addition, present in lye was a methoxy derivative of hydroxytyrosol, as assigned by means of its relative retention time to that of hydroxytyrosol, spectral data (*λ*_max_ at 239 nm and 267 nm as well as a shoulder at 389 nm) and published information [26], at the level of 110.5 mg/L. Regarding tyrosol, lye contained 75.5 mg/L, while the level of the compound in washing waters was 173.9 mg/L. RP-HPLC chromatogram recorded at UV 280 nm and 320 nm of both effluents exhibited also the presence of other phenolic compounds, including caffeic acid, luteolin-7-*O*-glucoside, and *p*-coumaric acid in lye (5.3 mg/L, 15.3 mg/L, and 19.2 mg/L, respectively) and washing waters (7.0 mg/L, 13.1 mg/L, and 20.7 mg/L, respectively). The non-phenolic secoiridoid compound oleoside-11-methyl ester was detected at UV 240 nm in the polar extract of washing waters at the level of 102.5 mg/L.

*B. trispora* growth in the non-enriched streams resulted in the decrease of hydroxytyrosol by 57.3% (58.0 mg/L) in lye and 66.8% (131.3 mg/L) in washing waters as well as in the complete degradation of *p*-coumaric acid in both effluents. Furthermore, the methoxy derivative of hydroxytyrosol present in lye decreased by 36.9% (69.7 mg/L). This finding highlights the ability of *B. trispora* to utilize the respective molecules at carbon-limited conditions in the streams (total soluble sugars: 6.1 g/L ± 0.2 g/L in lye and 5.2 g/L ± 0.1 g/L in washing waters). In contrast, the fungus could not degrade tyrosol as well as the other phenolic compounds, considering that non-significant changes were detected in their concentration at the end of the fermentation process. Thus, *B. trispora* shows low fungal selectivity for the degradation of these phenolic compounds. Overall, the degradation of hydroxytyrosol, methoxy derivative of hydroxytyrosol, and *p*-coumaric acid reflected in the decrease of the TPP content by 45.4% (226.4 mg/L ± 22.7 mg/L) in lye and 49.5% (361.5 mg/L ± 37.1 mg/L) in washing waters. Notably, non-significant changes were recorded in the content of TPP and individual phenolic compounds during the incubation of the non-inoculated effluents (< 7% reduction), verifying that *B. trispora* was responsible for the streams detoxification. The detoxification efficiency of *B. trispora* was important, although lower than the one reported for the fungal strain *A. niger* B60 in our previous study (up to 78% reduction in TPP content) [26]. Thus, the former species could be used as a bioremediation agent in single or mixed cultures to favor the subsequent treatment of the remaining contaminants.

Interestingly, none of the phenolic compounds in the nutrient-enriched lye were catabolized by the fungus, while only hydroxytyrosol was slightly degraded by 26.2% (291.9 mg/L) in the nutrient-enriched washing waters. As a consequence, the reduction of TPP content in the respective streams was 4.4% (396.6 mg/L ± 32.2 mg/L) and 14.5% (611.7 mg/L ± 43.1 mg/L). The above observations were attributed to the fact that glucose is the most easily assimilable carbon source for *B. trispora* growth and carotenogenesis in the nutrient-enriched effluents [9]. To our view, an obvious advantage can be marked using the enriched lye and washing water effluents for the development of a freshwater-free multi-product biorefinery process toward the simultaneous generation of products with high commercial value, i.e., *β*-carotene and bioactive phenolic compounds.

Overall, the proposed process for the production of *β*-carotene by *B. trispora* cultivated in lye and washing water effluents is described as follows. The effluents are enriched with nutrients, neutralized, and inoculated with *B. trispora* mated culture to start the fermentation process. After fermentation, the fungal biomass rich in *β*-carotene is separated from the remaining liquid (fermented effluents) and processed to recover *β*-carotene. The technical and economic benefits of the proposed valorization route are expected to increase if recovery of more than one target compound is designed [25]. This is the case of bioactive phenolic compounds recovery from the fermented effluents. The remaining streams after the recovery of the phenolic compounds are expected to have phytotoxic properties. Based on our previous study [26], this is mainly associated with the presence of Na in the effluents that is the primary inhibitor of seed germination and plant growth. Thus, subsequent treatment should be carried out for the removal of Na from the streams (e.g., diffusion dialysis and electrodialysis) [40] to enable their safe environmental disposal.

## 4. Conclusions

In conclusion, the data of the present study support the fact that the bioprocess for natural *β*-carotene production by *B. trispora* can be designed by implementing lye and washing waters from Spanish-style green olive processing as a sustainable water resource. The enrichment of these streams with nutrients ensures the ability of the fungus to synthesize notable quantities of the target carotenoid with high selectivity. These findings seem encouraging for further investigation. Specifically, of interest will be the enrichment of the wastewaters with nutrients from other agro-industrial wastes before recycling the former as feedstock for *β*-carotene fermentation by *B. trispora*. In addition, the study of a potential process scale-up together with the respective provision of process economics seems attractive. The stability of hydroxytyrosol and tyrosol content in the streams during carotenogenesis creates an additional source of input and boosts a sustainable design for potential multi-product biorefineries. From another point of view, inoculation of both effluents with the fungus in the absence of nutrients supports the development of efficient practices for the detoxification of table olive processing wastewaters. In the new era of the COVID-19 pandemic, the adoption of the above strategies toward the manufacturing of molecules (*β*-carotene, hydroxytyrosol, and tyrosol) with protective effects on human health is expected to stimulate the local economies and offer opportunities for public welfare.

## Figures and Tables

**Table 1:** Growth kinetic parameters (modified Logistic model) of *B. trispora* in non-sterile lye and washing waters without or after enrichment with nutrients as well as in synthetic medium (fermentation time: 6 days) ^1^.

Fermentation Substrate	Growth Parameters ^2^			Goodness of Fit
	*X*_m_ (g/L Broth)	*μ*_m_ (1/h)	*λ*_B_ (h)	*R* ^2^
Non-enriched lye	3.123 ^a^ ± 0.127	0.029 ^a^ ± 0.002	26.434 ^a^ ± 2.566	0.982 ^a^ ± 0.003
Nutrient-enriched lye	19.249 ^b^ ± 0.809	0.234 ^b^ ± 0.007	37.268 ^b^ ± 1.111	0.988 ^b,c^ ± 0.004
Non-enriched washing waters	0.830 ^c^ ± 0.048	0.012 ^c^ ± 0.002	45.554 ^c^ ± 1.350	0.989 ^b,c^ ± 0.004
Nutrient-enriched washing waters	13.776 ^d^ ± 0.137	0.223 ^b^ ± 0.010	10.435 ^d^ ± 1.364	0.994 ^c^ ± 0.002
Synthetic medium	10.007 ^e^ ± 0.355	0.172 ^d^ ± 0.008	14.319 ^e^ ± 0.364	0.987 ^a,b^ ± 0.001

^1^ Each value was expressed as mean ± standard deviation of three replicates. Different lowercase letters (a, b, c, d, and e) in the same column represent significant differences in values (*p*< 0.05). ^2^ *X*_m_ = maximum value of dry biomass content, *μ*_m_ = maximum specific growth rate, *λ*_B_ = growth lag time.

**Table 2:** Maximum total carotenoid and *β*-carotene production by *B. trispora* grown in non-sterile lye and washing waters without or after enrichment with nutrients as well as in synthetic medium (fermentation time: 6 days) ^1^.

Fermentation Substrate	Total Carotenoid Content		*β*-Carotene Content	
	mg/L Broth	mg/g Dry Biomass	mg/L Broth	mg/g Dry Biomass
Non-enriched lye	0.2 ^a^ ± 0.0	0.07 ^a^ ± 0.01	0.0 ^a^ ± 0.0	0.00 ^a^ ± 0.00
Nutrient-enriched lye	83.9 ^b^ ± 9.3	4.84 ^b^ ± 0.44	61.2 ^b^ ± 5.8	3.53 ^b^ ± 0.27
Non-enriched washing waters	0.1 ^a^ ± 0.0	0.08 ^a^ ± 0.01	0.0 ^a^ ± 0.0	0.01 ^a^ ± 0.00
Nutrient-enriched washing waters	83.5 ^b^ ± 11.7	5.95 ^c^ ± 0.95	64.1 ^b^ ± 8.7	4.57 ^c^ ± 0.71
Synthetic medium	56.5 ^c^ ± 10.0	5.51 ^b,c^ ± 0.68	44.8 ^c^ ± 6.3	4.39 ^c^ ± 0.37

^1^ Each value was expressed as mean ± standard deviation of three replicates. Different lowercase letters (a, b, and c) in the same column represent significant differences in values (*p*< 0.05).

**Table 3:** Proportion of *β*-carotene, *γ*-carotene, and lycopene at the maximum carotenoid production by *B. trispora* grown in non-sterile lye and washing waters after enrichment with nutrients as well as in synthetic medium (fermentation time: 6 days).^1.^

Fermentation Substrate	% of Total Carotenoid Content		
	*β*-Carotene	*γ*-Carotene	Lycopene
Nutrient-enriched lye	81.6 ^a^ ± 0.1	9.1 ^a^ ± 0.1	9.3 ^a^ ± 0.1
Nutrient-enriched washing waters	87.6 ^b^ ± 0.3	4.8 ^b^ ± 0.3	7.6 ^b^ ± 0.1
Synthetic medium	89.8 ^c^ ± 0.2	2.6 ^c^ ± 0.1	7.6 ^b^ ± 0.2

^1^ Each value was expressed as mean ± standard deviation of three replicates. Different lowercase letters (a, b, and c) in the same column represent significant differences in values (*p* < 0.05).

**Table 4:** *β*-Carotene production kinetic parameters (modified Logistic model) during *B. trispora* growth in non-sterile lye and washing waters after enrichment with nutrients as well as in synthetic medium (fermentation time: 6 days) ^1^.

Fermentation Substrate	*β*-Carotene Production Parameters ^2^			Goodness of Fit
	*P*_m_ (mg/L Broth)	*R*_m_ (1/h)	*λ*_CR_ (h)	*R* ^2^
Nutrient-enriched lye	83.032 ^a^ ± 0.798	2.018 ^a^ ± 0.069	47.320 ^a^ ± 0.524	0.999 ^a^ ± 0.001
Nutrient-enriched washing waters	81.494 ^a^ ± 1.765	1.418 ^b^ ± 0.048	31.052 ^b^ ± 0.828	0.994 ^b^ ± 0.001
Synthetic medium	50.655 ^b^ ± 0.182	0.842 ^c^ ± 0.044	26.221 ^c^ ± 1.012	0.978 ^c^ ± 0.005

^1^ Each value was expressed as mean ± standard deviation of three replicates. Different lowercase letters (a, b, and c) in the same column represent significant differences in values (*p*< 0.05). ^2^ *P*_m_ = maximum value of *β*-carotene content, *R*_m_ = maximum specific *β*-carotene production rate, *λ*_CR_ = *β*-carotene production lag time.

**Table 5:** Content of individual phenolic/secoiridoid compounds in non-sterile lye (non-enriched or nutrient-enriched) prior and after fermentation at the maximum *β*-carotene production by *B. trispora* (fermentation time: 6 days).

Compound ^1^	Concentration (mg/L) ^2^			*λ*_max_ (nm) ^3^	FL ^4^
	0 d	6 d (Non-Enriched)	6 d (Enriched)		
HTyr	135.9 ^a^ ± 5.5	58.0 ^b^ ± 8.0	131.3 ^a^ ± 10.9	239, 279	yes
OMeHTyr	110.5 ^a^ ± 3.9	69.7 ^b^ ± 5.8	100.1 ^a^ ± 7.5	239, 267, 389	no
Tyr	75.5 ^a^ ± 2.8	72.6 ^a^ ± 8.0	73.1 ^a^ ± 6.5	239, 275	yes
CA	5.3 ^a^ ± 0.3	5.1 ^a^ ± 0.4	5.2 ^a^ ± 0.3	241, 289, 322	no
LuG	15.3 ^a^ ± 0.6	14.9 ^a^ ± 1.3	15.1 ^a^ ± 1.0	245, 348	no
CuA	19.2 ^a^ ± 0.8	not detected	19.5 ^a^ ± 1.6	242, 307	no

^1^ HTyr = hydroxytyrosol, OMeHTyr = methoxy derivative of hydroxytyrosol, Tyr = tyrosol, CA = caffeic acid, LuG = luteolin-7-*O*-glucoside, and CuA = *p*-coumaric acid. ^2^ Each value was expressed as mean ± standard deviation of three replicates. Different lowercase letters (a and b) in the same row represent significant differences in values (*p*< 0.05). ^3^ *λ*_max_ = maximum absorbance (UV spectra). ^4^ FL = fluorescence.

**Table 6:** Content of individual phenolic/secoiridoid compounds in non-sterile washing waters (non-enriched or nutrient-enriched) prior and after fermentation at the maximum *β*-carotene production by *B. trispora* (fermentation time: 6 days).

Compound ^1^	Concentration (mg/L) ^2^			*λ*_max_ (nm) ^3^	FL ^4^
	0 d	6 d (Non-Enriched)	6 d (Enriched)		
HTyr	395.7 ^a^ ± 18.4	131.3 ^b^ ± 15.8	291.9 ^c^ ± 26.1	239, 279	yes
Tyr	173.9 ^a^ ± 6.8	167.9 ^a^ ± 17.6	170.1 ^a^ ± 13.0	239, 275	yes
OME	102.5 ^a^ ± 4.0	99.0 ^a^ ± 8.4	100.8 ^a^ ± 7.4	241	no
CA	7.0 ^a^ ± 0.3	6.7 ^a^ ± 0.6	6.9 ^a^ ± 0.4	241, 289, 322	no
LuG	13.1 ^a^ ± 0.6	12.5 ^a^ ± 1.0	12.8 ^a^ ± 1.2	245, 348	no
CuA	20.7 ^a^ ± 1.0	not detected	19.9 ^a^ ± 2.0	242, 307	no

^1^ HTyr = hydroxytyrosol, Tyr = tyrosol, OME = oleoside-11-methyl ester, CA = caffeic acid, LuG = luteolin-7-*O*-glucoside, and CuA = *p*-coumaric acid. ^2^ Each value was expressed as mean ± standard deviation of three replicates. Different lowercase letters (a, b, and c) in the same row represent significant differences in values (*p* < 0.05). ^3^ *λ*_max_ = maximum absorbance (UV spectra). ^4^ FL = fluorescence.
